# Single‐cell and bulk characterisation of the distinct immune landscape and possible regulatory mechanisms in coronary plaques vulnerability

**DOI:** 10.1002/ctm2.1281

**Published:** 2023-06-14

**Authors:** Bo Liang, Wei‐Lin Liang, Hui‐Ling Liao

**Affiliations:** ^1^ Department of Nephrology, The Key Laboratory for the Prevention and Treatment of Chronic Kidney Disease of Chongqing, Chongqing Clinical Research Center of Kidney and Urology Diseases, Xinqiao Hospital Army Medical University (Third Military Medical University) Chongqing China; ^2^ Department of Cardiology Guangyuan Hospital of Traditional Chinese Medicine Guangyuan China; ^3^ The Affiliated Traditional Chinese Medicine Hospital of Southwest Medical University Luzhou China; ^4^ College of Integrated Traditional Chinese and Western Medicine Southwest Medical University Luzhou China

To the Editor:

Atherosclerosis is an immune disease that can lead to the formation of atherosclerotic plaques.^1^ When atherosclerotic plaques become fragile or unstable, they can rupture and, in severe cases, lead to myocardial infarction or stroke.^2^ The immunologically relevant genes play essential roles in immune infiltration.^3^ A previous study revealed the obvious characteristics of immune cells in carotid plaque of patients with clinical symptomatic disease compared to asymptomatic disease.^4^ The characteristics of immune‐related genes, however, and their regulatory mechanisms in coronary plaques vulnerability are still unclear. Here, we compared the heterogeneity of immune cells in coronary plaques from acute coronary syndrome (ACS) and stable angina pectoris (SAP) patients, corresponding to unstable and stable plaques, respectively, through single‐cell transcriptomic data. Moreover, important results were validated in the bulk transcriptomic data, and we preliminary explored the transcription factor‐related regulatory mechanisms.

The detailed methods are described in the Supporting Information. The single‐cell profiling of immune cells in coronary plaque obtained from GSE184073^5^ is shown in Figure S1, and we clustered five cell types, namely T cells, B cells, monocytes, NK cells and macrophages (Figure 1A,B). The SAP group has more T cells, while the ACS group has more monocytes (Figure 1C). In addition, macrophages, T cells and NK cells exhibited higher purity, whereas B cells and monocytes showed higher heterogeneity (Figure 1D). To further investigate variations in the regulatory network of coronary plaque, we used hallmark gene sets to assess differences in the pathways between the ACS and SAP groups. Intriguingly, monocytes, macrophages and T cells showed upregulation of a wide variety of pathway activities, including different aspects of immunology, metabolism, signalling and proliferation (Figure 1E), implying that coronary plaques' vulnerability may preferentially remodel these cells and induce specific functional states.

**FIGURE 1:**
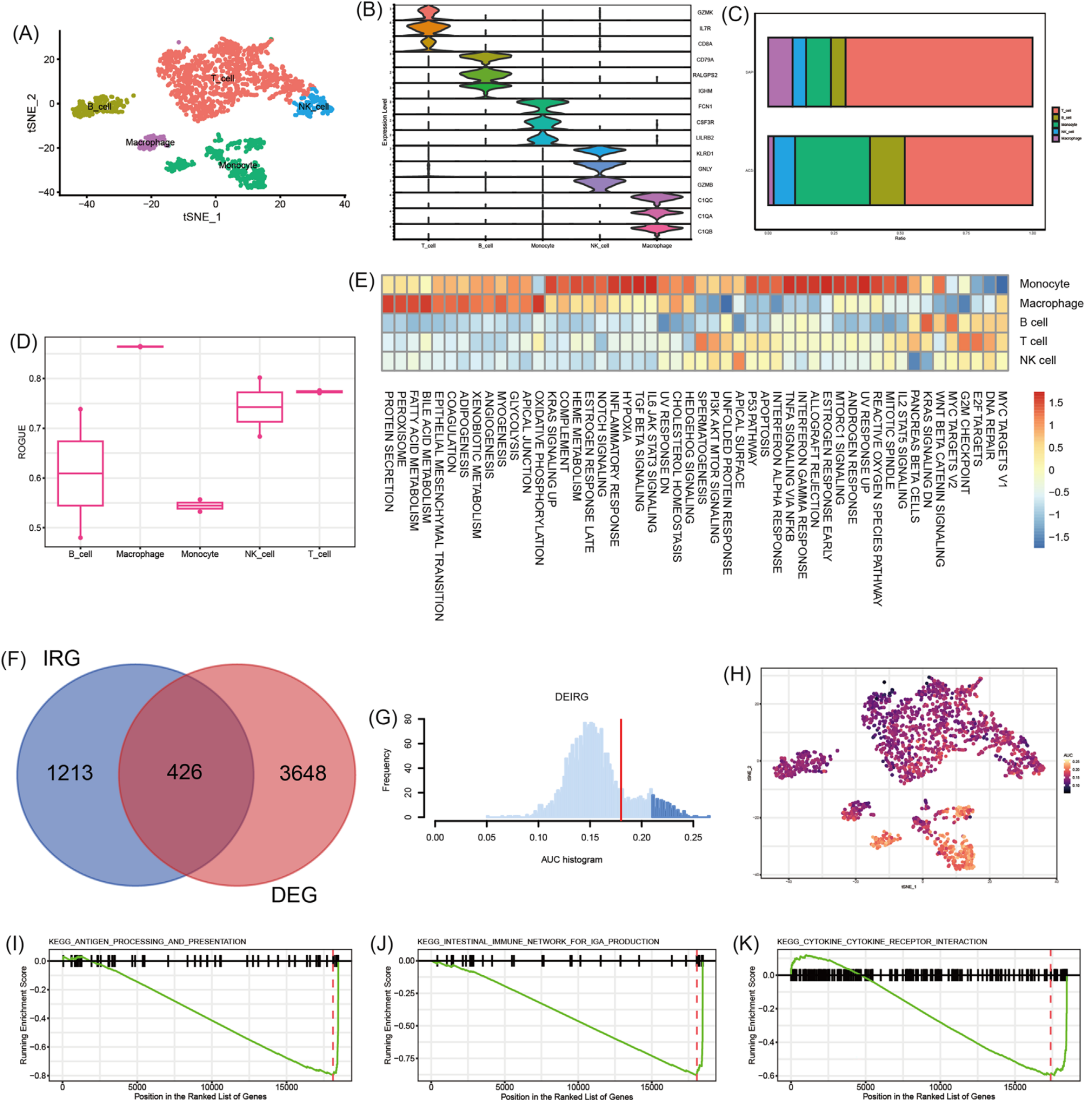
Dissection of the immune landscape and differentially expressed immunologically relevant genes (DEIRG) scores of immune cell clusters in coronary plaques. (A) t‐SNE projection of 1993 cells from the pooled coronary plaques and different cell types were coloured with unique colours. (B) Stacked violin plot depicted distributions of cell type marker genes in each cell type cluster using density curves. (C) Cell type cluster distribution in the ACS and SAP groups. (D) Boxplot showing cell purity for each cell type by ROGUE. (E) Gene set variation analysis in the corresponding cell populations. (F) Venn diagram of screened DEIRGs. (G) Score of 426 screened DEIRGs. The threshold was chosen as 0.24. (H) t‐SNE plots of DEIRG score in all cell type clusters. (I) Antigen processing and presentation in monocytes. (J) Intestinal immune network for IgA production. (K) Cytokine and cytokine receptor interaction in T cells.

We achieved 426 differentially expressed immunologically relevant genes (DEIRGs) (Figure 1F). We then determined the DEIRGs activity (Figure 1G) and found that monocytes and T cells expressed more genes (Figure 1H). As these cells were remarkably dysregulated in the ACS group (Figure 1C), we further conducted the functional analysis of differentially expressed genes (DEGs) in the T cells and monocyte clusters from immune cell clusters in the coronary plaque and we enriched antigen processing and immune response (Figure 1I–K) and some classical signalling pathways (Figure S2). The top 15 results of upregulated and downregulated DEGs in monocytes and T cells are shown in Figure S3A–H, which were mostly related to immune response.

Then we reconstructed the differentiation trajectory. Through uniform manifold approximation and projection dimensionality reduction, we cannot obtain several T‐cell clusters due to limited cells (Figure 2A and Figure S4A). Then we reconstructed the cell developmental relationships, and we found differentiation stages (Figure 2B). Both the number (ligand–receptor pairs) and strength of cell–cell communication were high (Figure 2C,D and Figure S4C,D). We detected 40 significant pathways between five immune cell clusters in the coronary plaque, and the C‐C Motif Chemokine Ligand (CCL) signalling pathway exhibited the most signalling patterns, and T cell cluster exhibited the most signalling pathways (Figure 2E). Moreover, we found the largest number of ligand–receptor pairs sent from the T cells cluster to NK cells clusters (Figure S4E). Furthermore, the T cells cluster exhibited high expression in the CCL signalling pathway (Figure 2F–H) and the most significant ligand–receptor pairs were CCL5–CCR1 (Figure 2I and Figure S4F).

**FIGURE 2:**
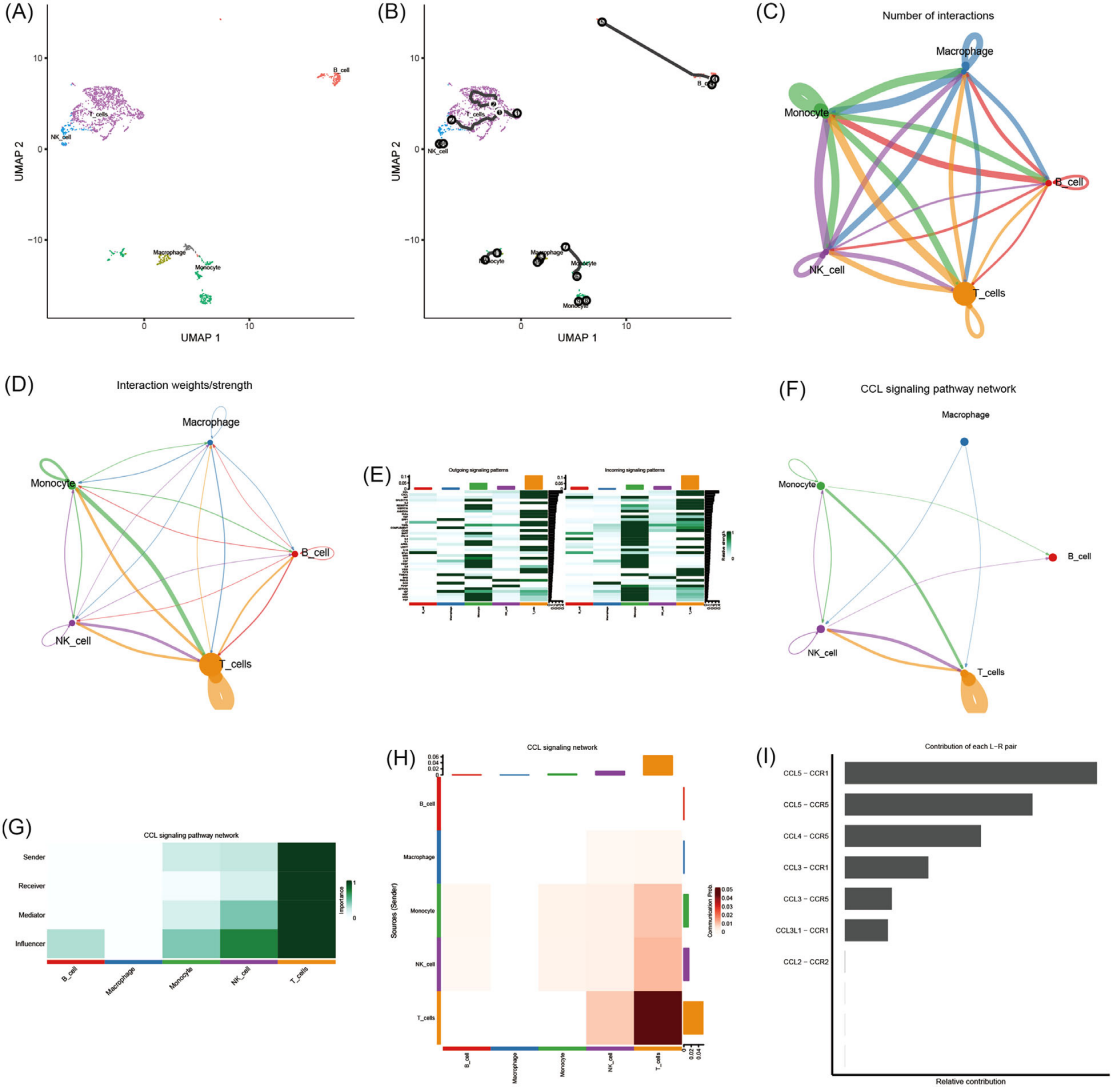
Cell developmental relationships and communication. (A) Uniform manifold approximation and projection dimensionality reduction. (B) Two‐dimensional cell trajectory. (C) Number of interactions. The size of the circles of various colours in the periphery indicated the number of cells, the larger the circle, the more the number of cells; the cells that emit arrows expressed ligands, and the cells pointed to by the arrows expressed receptors, and the more ligand–receptor pairs, the thicker the line. (D) Interaction weights/strength. Strength was the sum of weights. (E) Relative strength of each signalling pathway network for each cell type cluster with both incoming and outgoing signalling patterns. (F) Inferred CCL signalling pathway network. (G) Relative importance of each cell type cluster ranked according to the computed four network centrality measures in the CCL signalling network display. (H). Relative importance of each cell type cluster ranked according to the computed sender source in the CCL signalling network displaying. The *x*‐axis was the cells that emitted the signal, the *y*‐axis was the cell that received the signal, the heatmap represented the communication possibility according to the colour shade, the upper and right columns were the accumulation of the *y*‐axis and the *x*‐axis. (I) Relative contribution of each ligand–receptor pair as it affects the overall communication network of the CCL signalling pathway.

Later, we used the bulk transcriptomic data to validate our results. A total of 1010 and 6373 dysregulated DEGs were retained in human peripheral blood mononuclear cells in GSE59867 and GSE62646, respectively (Figure 3A,B). The functional analysis of DEGs in coronary plaques interestingly focused on antigen processing and immune response (Figure 3C,D), which is consistent with the previous characterisation of the monocytes and T‐cell clusters from immune‐cell clusters in coronary plaque. As immune response was focused by functional enrichment analysis of DEGs from the monocytes and T cells clusters and bulk DEGs, we further investigated the common expression characteristics of DEIRGs between the monocytes and T‐cell clusters from the coronary plaques and human peripheral blood mononuclear cells. A total of 39 common DEIRGs were identified in both the different cell type clusters from the coronary plaques and human peripheral blood mononuclear cells (Figure 3E). Expression of these common DEIRGs was mainly dysregulated in the different cell type clusters from the coronary plaques (Figure 3F and Figure S5A). Furthermore, 19 common differentially expressed transcription factors (DETFs) were differently expressed in both T cells and monocytes clusters from human peripheral blood mononuclear cells and coronary plaques (Figure 3G). The expressions of these common DETFs are shown in Figure 3H,I and Figure S5B. All 19 DETFs were active in the monocytes cluster (Figure 3H) and 18 DETFs were upregulated in the human peripheral blood mononuclear cells (Figure 3I). Finally, the protein–protein interaction network indicated that CEBPB and SPI1 may play a key role in the possible regulatory mechanisms as a hub transcription factor (Figure 3J,K).

**FIGURE 3:**
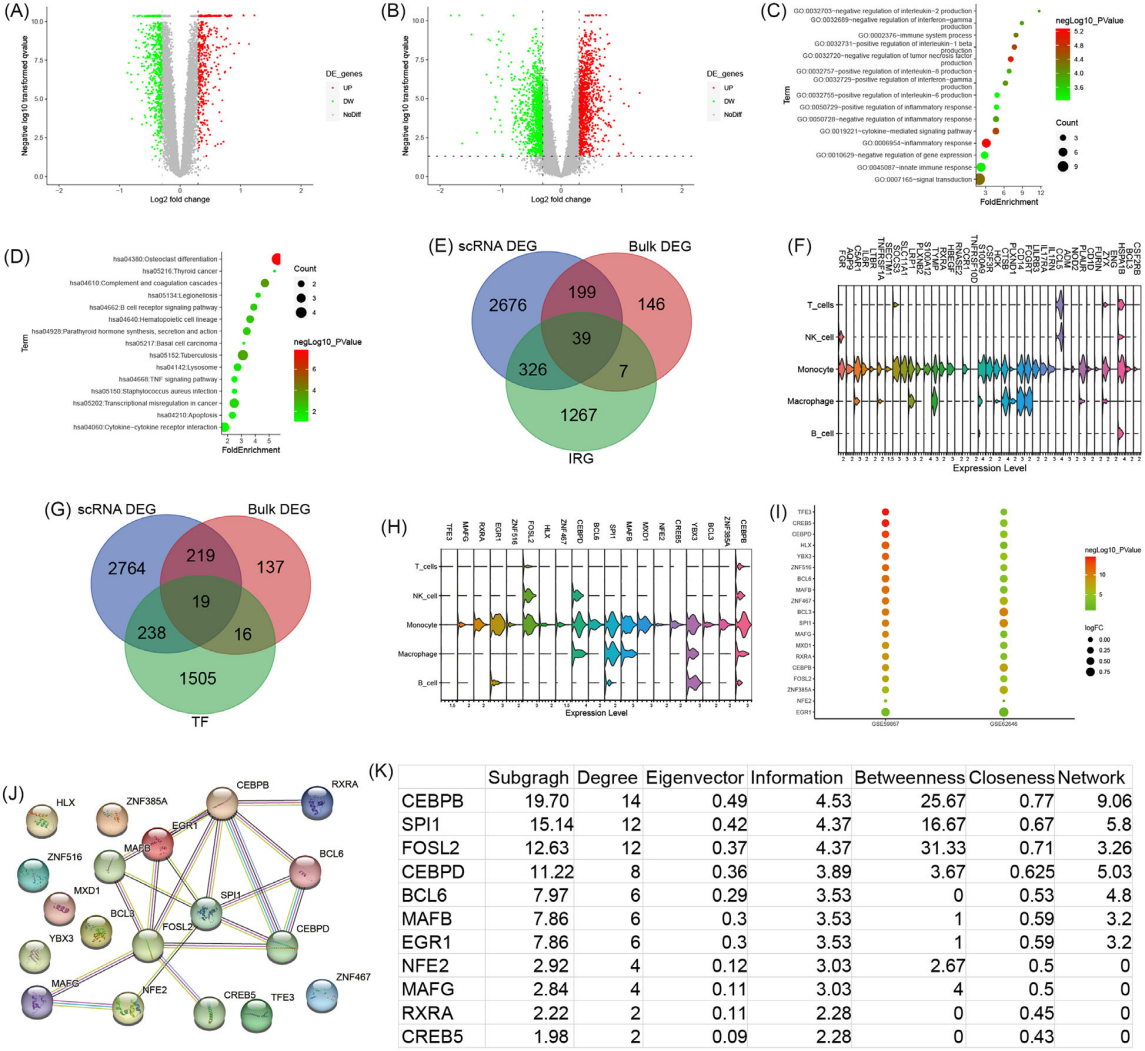
Bulk differentially expressed genes (DEGs) of human peripheral blood mononuclear cells and common differentially expressed immunologically relevant genes (DEIRGs) and transcription factors regulatory mechanisms. (A) Volcano plot of DEGs in the GSE59867 dataset. (B) Volcano plot of DEGs in the GSE62646 dataset. (C) BP of bulk DEGs. (D) KEGG of bulk DEGs. (E) Venn plot showed the common DEIRGs. (F) Stacked violin plot of 39 common DEIRGs in immune cells from the coronary plaque. (G) Venn plot showed the common differentially expressed transcription factors (DETFs). (H) Stacked violin plot of nine common DETFs in immune cells from the coronary plaque. (I) The expression of 19 common DETFs in human peripheral blood mononuclear cells. (J) The PPI network of 19 common DETFs illustrated using STRING. (K) Results of CytoNCA ranked by Subgragh.

Single‐cell RNA sequencing is an ideal method to map the cellular and molecular composition of atherosclerotic plaque and can help to find new precise immunotherapies.^6^, ^7^ Because the plaques are derived from the carotid artery, the mechanisms of coronary atherosclerosis may not be fully elucidated. We present here data from coronary plaques to complement the study of atherosclerosis (Figure 4). We used a 10× genomics single‐cell RNA sequencing platform to characterise the CD45^+^ immune cells from the coronary plaques from the patients with ACS and SAP. A total of five immune cell clusters were distinguished and monocytes and T cells were considered as the targets for immunotherapies. We also validated our results in the bulk transcriptomic data and preliminary explored the transcription factor‐related regulatory mechanisms. In conclusion, we used relatively standard analysis pipelines to uncover immune cells subpopulations and their transcriptomic signatures in patients with stable and unstable coronary plaques with some preliminary validation of selected clusters and mechanism exploration of transcriptional regulation. Cross‐talk between monocytes and T cells may enhance plaques vulnerability and CEBPB is involved in the transcriptional regulation of atherosclerosis. Our findings provide important information for understanding the cellular and molecular mechanisms of coronary plaques vulnerability and may contribute to the atherosclerotic therapy.

**FIGURE 4:**
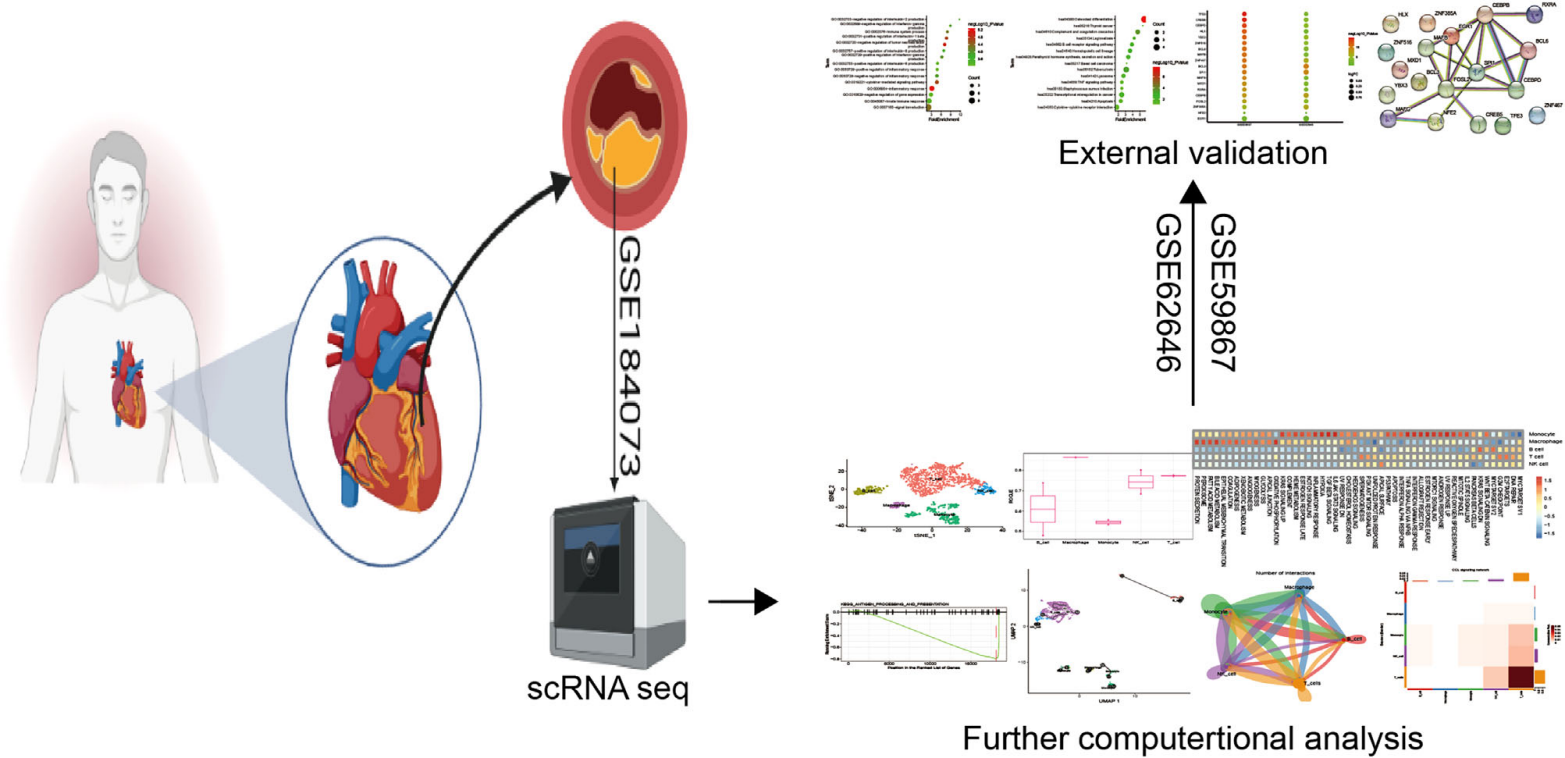
Schematic of the overall study design.

## CONFLICT OF INTEREST STATEMENT

The authors declare no conflicts of interest.

## Supporting information

Supporting Information

Supporting Information
