# Supplementary material for: Single‐cell and bulk characterisation of the distinct immune landscape and possible regulatory mechanisms in coronary plaques vulnerability
Source: Clin Transl Med. 2023 Jun 14;13(6):e1281. doi: 10.1002/ctm2.1281 (PMC10265436; doi:10.1002/ctm2.1281)
Supplement: Supplementary file 1 — Supporting Information [file CTM2-13-e1281-s001.pdf]

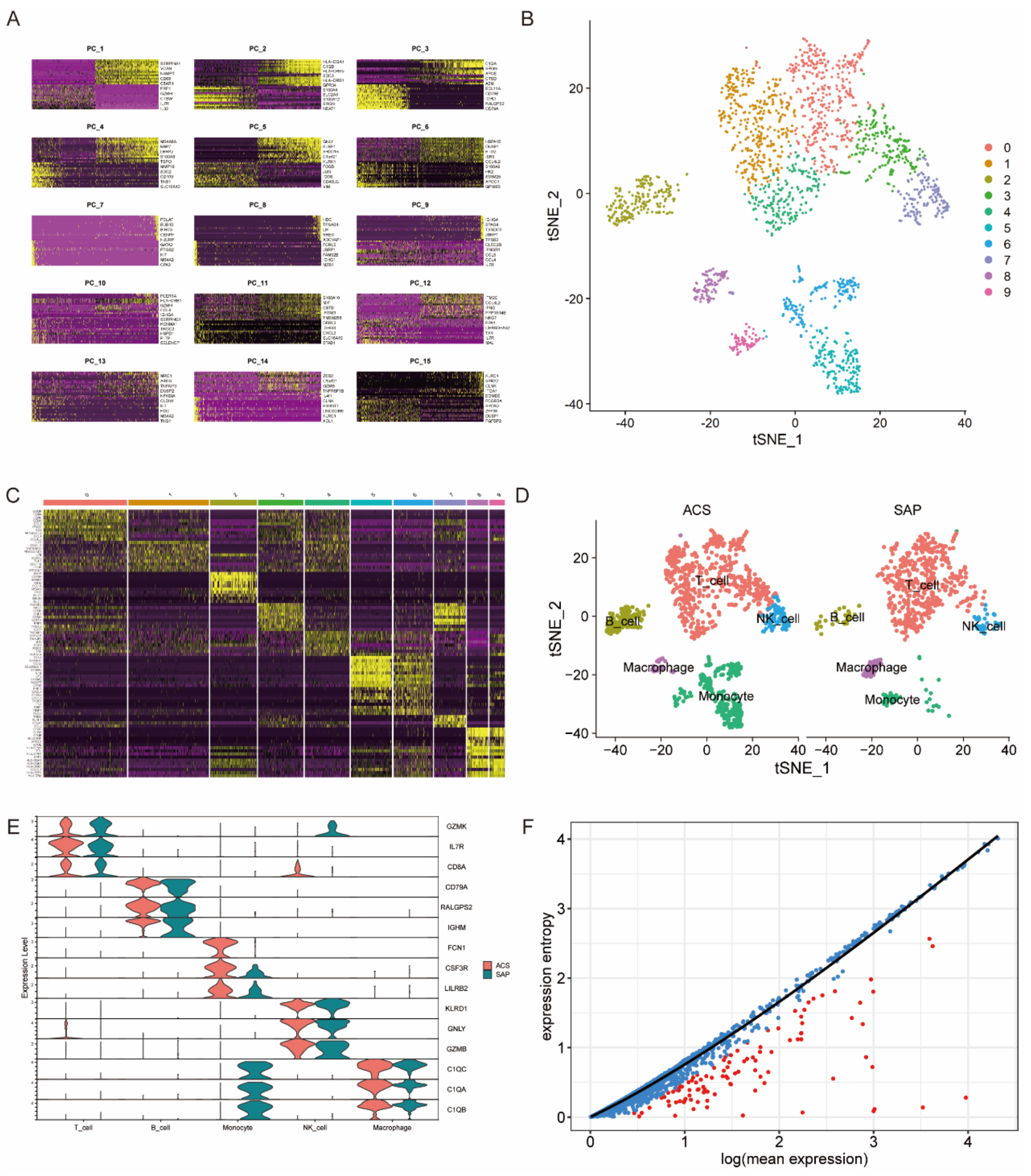

Figure S1. scRNA analysis of coronary plaque. A. Heatmap of top 10 DEGs in each PCA component. B. Cells were identified to ten clusters. C. Heatmap of top 10 DEGs in each cluster and the top 10 DEGs were labeled in yellow color. D. t-SNE projection of the ACS and SAP groups, respectively. E. Stacked violin plot depicted distributions of cell type marker genes in the ACS and SAP groups. F. S-E plot of the identified highly informative genes.

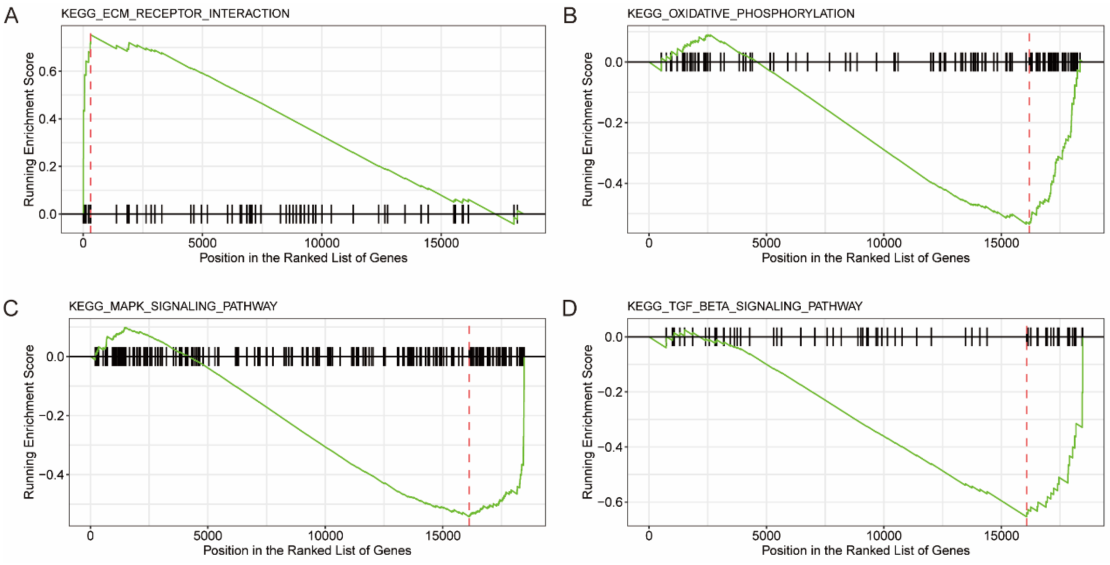

Figure S2. Gene set enrichment analysis of DEGs in the T cells and monocytes clusters from immune cell clusters in the coronary plaque. A. ECM receptor interaction in monocytes. B. Oxidative phosphorylation in monocytes. C. MAPK signaling pathway in T cells. D. TGF beta signaling pathway in T cells.

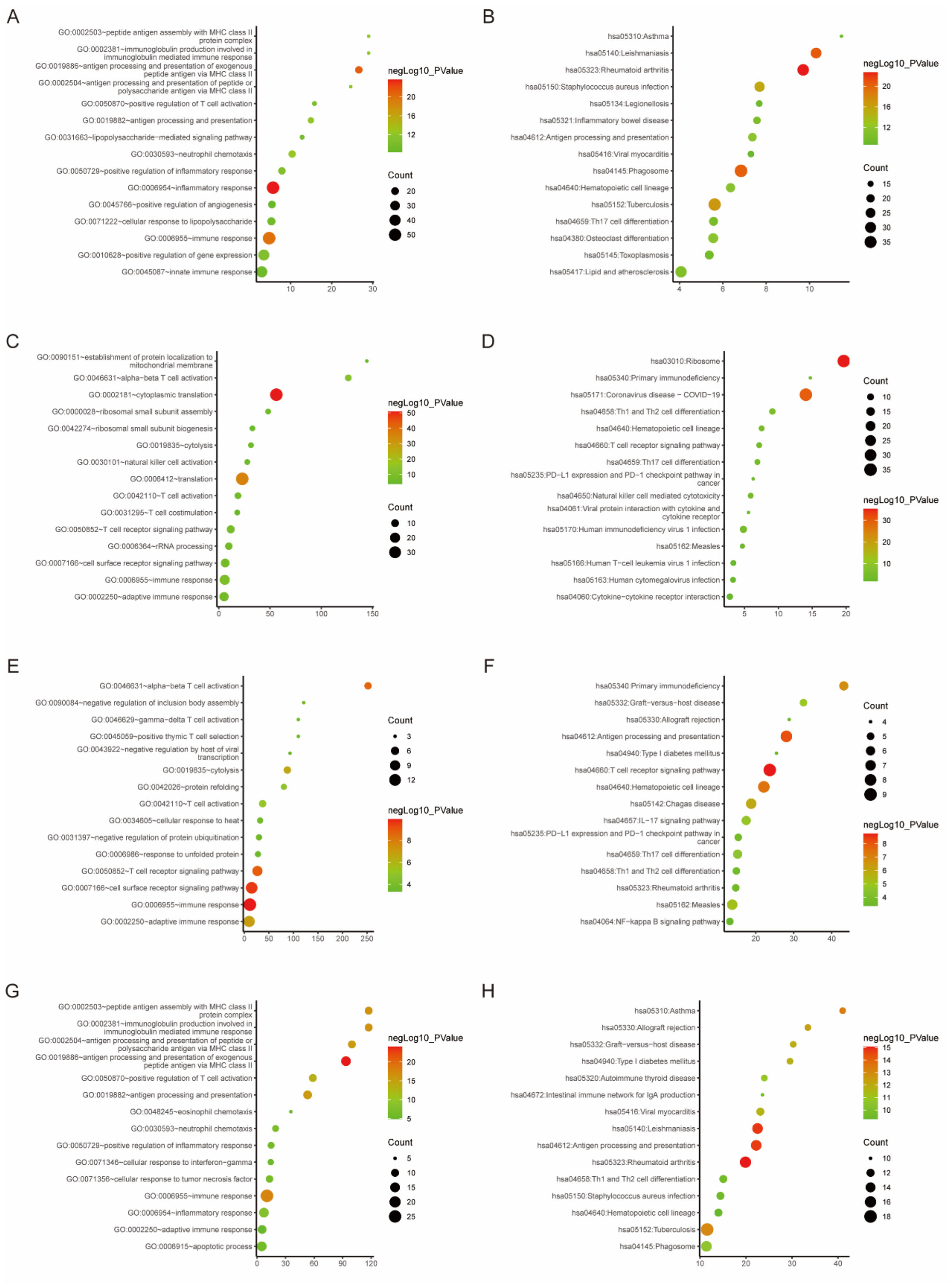

Figure S3. Functional enrichment analysis of DEGs in monocytes and T cells in coronary plaque. A. BP analysis of upregulated DEGs in monocytes. B. KEGG of upregulated DEGs in monocytes. C. BP analysis of downregulated DEGs in monocytes. D. KEGG of downregulated DEGs in monocytes. E. BP analysis of upregulated DEGs in T cells. F.

KEGG of upregulated DEGs in T cells. G. BP analysis of downregulated DEGs in T cells.

H. KEGG of downregulated DEGs in T cells.

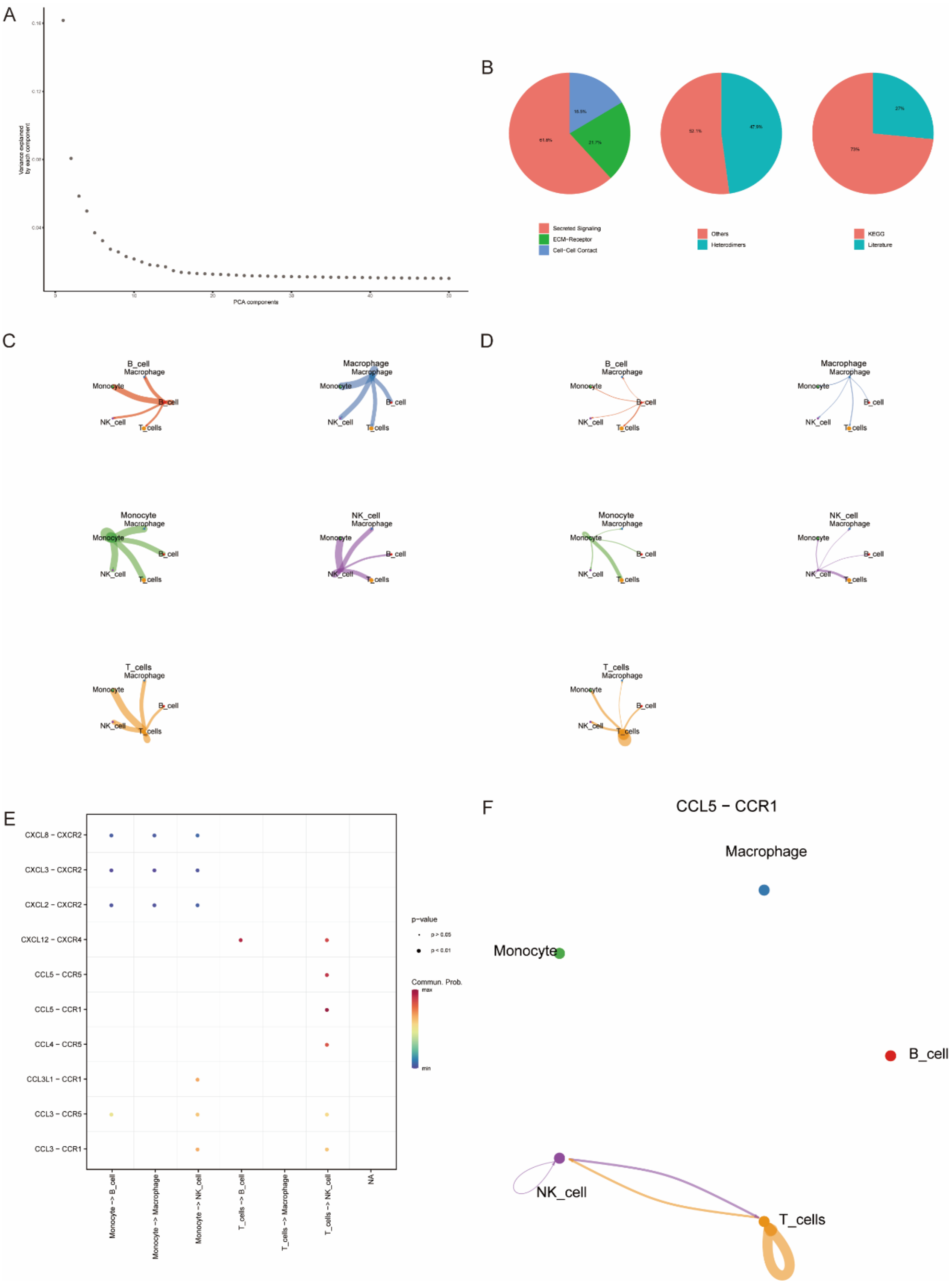

Figure S4. Cell developmental relationships and communication. A. Variance explained by each PCA component. B. Categories in the CellChat database. C. Number of

interactions in each cell type. The size of the circles of various colors in the periphery indicated the number of cells, the larger the circle, the more the number of cells; the cells that emit arrows expressed ligands, and the cells pointed to by the arrows expressed receptors, and the more ligand-receptor pairs, the thicker the line. D. Interaction weights/strength in each cell type. Strength was the sum of weights. E. Regulation different immune cells clusters. F. Relative strength of CCL5-CCR1 ligand-receptor pair network for each cell type cluster with both incoming and outgoing signaling patterns.

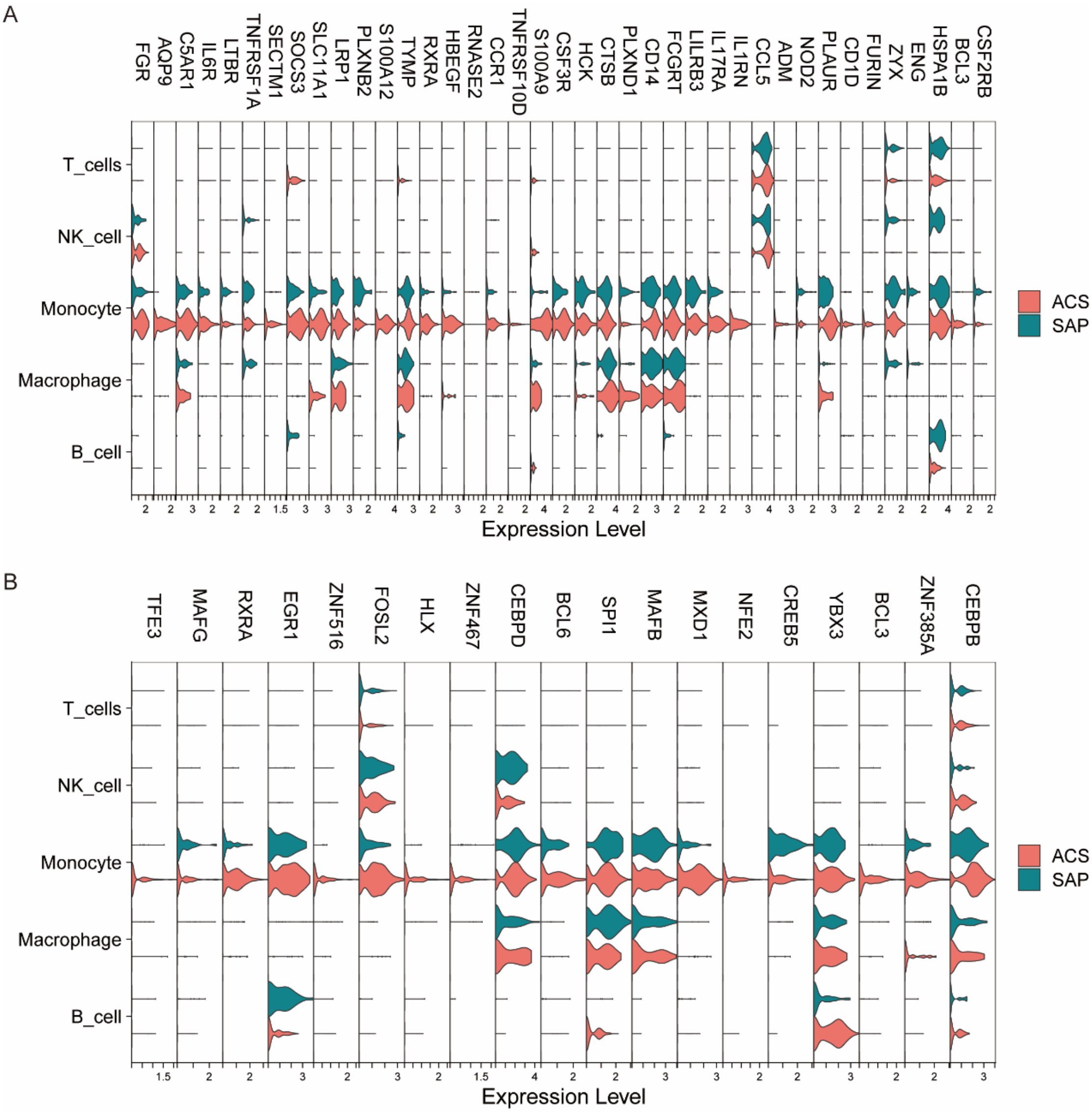

Figure S5. The expression of common DEIRGs and common DETFs in the immune cells

from the coronary plaques regardless the ACS and SAP groups. A. Common DEIRGs. B. Common DETFs.
