# Supplementary material for: Single‐cell and bulk characterisation of the distinct immune landscape and possible regulatory mechanisms in coronary plaques vulnerability
Source: Clin Transl Med. 2023 Jun 14;13(6):e1281. doi: 10.1002/ctm2.1281 (PMC10265436; doi:10.1002/ctm2.1281)
Supplement: Supplementary file 2 — Supporting Information [file CTM2-13-e1281-s002.pdf]

Supplemental Material---Methods

1 Single-cell transcriptomic data processing

Four patients with chronic coronary syndrome (SAP) without high-intensity signals in coronary plaques on non-contrast T1-weighted magnetic resonance imaging and three patients with ACS were projected with directional coronary atherectomy to obtain the pooled coronary plaque. The investigation conformed to the principles outlined in the Declaration of Helsinki and written informed consent was provided by all participants or their legal representatives. $CD45^{+}$ immune cells were sorted from the pooled plaques and applied to the 10X platform[1]. The feature-barcode gene expression matrix was obtained from GSE184073 (GPL24676 Illumina NovaSeq 6000 (Homo sapiens)) and then analyzed with the *Seurat* package (version 4.1.0)[2]. Cells expressing > 500 genes and < 4000 genes and < 20% mitochondrial genes were initially included. Filtered cells were normalized with the *LogNormalize* method and 2000 most variable genes were selected with the *vst* method. Subsequently, principal component analysis (PCA) was then performed on the variable genes, and significant PCA components were selected. Then the P value distribution was visualized using the *JackStraw* and *ScoreJackStraw* functions. The *FindNeighbors* and *FindClusters* functions with a resolution of 0.5 were used to cluster the cells into different clusters, and the t-distributed stochastic neighbor embedding (t-SNE) analysis was conducted. The *FindAllMarkers* function with min.pct of 0.25 and logfc.threshold of 0.25 was applied to identify differentially expressed genes (DEGs) for each cluster. Cell types were identified based on the DEGs in each cluster and checked according to HumanPrimaryCellAtlasData with the *celldex* package (version

1.4.0)[3]. The scores within cells and delta distribution were used to evaluate the cell type annotation results. Finally, the cell type distribution in each group (the ACS and SAP groups) was calculated to find the differential cell types.

2 Heterogeneity assessment

ROGUE package (version 1.0)[4] was utilized with the default parameter settings for recommended pipelines to accurately quantify the purity of identified cell clusters.

3 Gene set variation and enrichment analysis

Gene set variation analysis with *GSVA* package (version 1.42.0)[5] was used to analyze the functional differences in the corresponding cell populations and gene set enrichment analysis was used to assess the pathway-enrichment status[6].

4 Differentially expressed immunologically relevant gene (DEIRG) score

An immunologically relevant list of genes curated with functions and Gene Ontology terms was obtained from ImmPort[7]. When this list was intersected with the DEGs, we obtained the DEIRGs. The DEIRG score was calculated with the *AUCell* package (version 1.16.0)[8].

5 Trajectory analysis

The differentiation trajectories of most abundant cells were inferred with the *monocle3* package (version 1.0.0)[9]. Data were preprocessed with the PCA method and dimensionality reduction was employed using the uniform manifold approximation and projection method. Finally, two-dimensional trajectories were presented.

6 Cell-cell communication analysis

The intercellular communication analysis was inferred, analyzed, and visualized with the

CellChat package (version 1.4.0)[10]. Further, we only selected cells that were present in the SAP and ACS groups for analysis.

7 Bulk transcriptomic data processing

Bulk transcriptomic data of human peripheral blood mononuclear cells isolated from patients with ST-segment elevation myocardial infarction (STEMI) on the 1st day of myocardial infarction and stable coronary artery disease and without a history of myocardial infarction from GSE59867[11] and GSE62646[12] were analyzed in the present study. GSE59867 (GPL6244 [HuGene-1_0-st] Affymetrix Human Gene 1.0 ST Array [transcript (gene) version]) comprised 111 STEMI patients and 46 stable coronary artery disease patients and GSE62646 (GPL6244 [HuGene-1_0-st] Affymetrix Human Gene 1.0 ST Array [transcript (gene) version]) included 28 STEMI patients and 14 stable coronary artery disease patients. Raw data was downloaded using the *GEOquery* package (version 2.62.0)[13] and DEGs were calculated using the *limma* package (version 3.50.1)[14]. The Bulk DEG was obtained by taking the intersection of the DEGs with the most significant difference (an adjusted P value <0.05, and an absolute logFoldChange > 0.3) between the two datasets.

8 Functional enrichment analysis

The DEGs from the different cell types clusters and Bulk DEG were independently imported into DAVID 2021[15] for Gene Ontology-biological process (BP) and Kyoto Encyclopedia of Genes and Genomes (KEGG) analysis. The *ggplot2* package (version 3.3.5) was conducted for visualization, as described previously[16].

9 Common DEIRGs and transcription factors regulatory mechanisms

The DEGs from the different cell types clusters and human peripheral blood mononuclear cells (bulk DEGs) and immune-related genes were analyzed to obtain the common DEIRGs. Then the expression of the common DEIRGs in the immune cells sorted from the pooled coronary plaques was shown.

All human transcription factors were downloaded from hTFtarget[17] and AnimalTFDB (version 3.0)[18]. The DEGs from the differential cell types clusters and Bulk DEG converged with all human transcription factors to obtain the differentially expressed transcription factors (DETF). Then the expressions of these DETFs in the immune cells sorted from the pooled coronary plaques and human peripheral blood mononuclear cells were calculated. Finally, the protein-protein interaction network analysis was performed using STRING (version 11.5)[19] to explore the transcription factors' regulatory mechanisms in coronary plaque vulnerability, as described previously[20]. The CytoNCA plugin (version 2.1.6)[21] in Cytoscape (version 3.9.1)[22] was used to evaluate the multiple centralities in the protein-protein interaction network.

References

1. Emoto T, Yamamoto H, Yamashita T, et al. Single-Cell RNA Sequencing Reveals a Distinct Immune Landscape of Myeloid Cells in Coronary Culprit Plaques Causing Acute Coronary Syndrome. *Circulation*. 2022/05/03 2022;145(18):1434-1436. doi:10.1161/CIRCULATIONAHA.121.058414

2. Hao Y, Hao S, Andersen-Nissen E, et al. Integrated analysis of multimodal single-cell

data. *Cell*. 2021;184(13):3573-3587.e29. doi:10.1016/j.cell.2021.04.048

3. Aran D, Looney AP, Liu L, et al. Reference-based analysis of lung single-cell sequencing reveals a transitional profibrotic macrophage. *Nat Immunol*. 2019;20(2):163-172. doi:10.1038/s41590-018-0276-y

4. Liu B, Li C, Li Z, Wang D, Ren X, Zhang Z. An entropy-based metric for assessing the purity of single cell populations. *Nat Commun*. Jun 22 2020;11(1):3155. doi:10.1038/s41467-020-16904-3

5. Hänzelmann S, Castelo R, Guinney J. GSVA: gene set variation analysis for microarray and RNA-seq data. *BMC Bioinformatics*. 2013;14:7. doi:10.1186/1471-2105-14-7

6. Subramanian A, Tamayo P, Mootha VK, et al. Gene set enrichment analysis: A knowledge-based approach for interpreting genome-wide expression profiles. *Proceedings of the National Academy of Sciences*. 2005;102(43):15545-15545. doi:10.1073/pnas.0506580102

7. Bhattacharya S, Dunn P, Thomas CG, et al. ImmPort, toward repurposing of open access immunological assay data for translational and clinical research. *Scientific data*. 2018;5:180015-180015. doi:10.1038/sdata.2018.15

8. Aibar S, González-Blas CB, Moerman T, et al. SCENIC: single-cell regulatory network inference and clustering. *Nat Methods*. 2017;14(11):1083-1086. doi:10.1038/nmeth.4463

9. Cao J, Spielmann M, Qiu X, et al. The single-cell transcriptional landscape of mammalian organogenesis. *Nature*. 2019;566(7745):496-502. doi:10.1038/s41586-019-0969-x

10. Jin S, Guerrero-Juarez CF, Zhang L, et al. Inference and analysis of cell-cell communication using CellChat. *Nat Commun*. 2021;12(1):1088-1088.

doi:10.1038/s41467-021-21246-9

11. Maciejak A, Kiliszek M, Michalak M, et al. Gene expression profiling reveals potential prognostic biomarkers associated with the progression of heart failure. *Genome Med*. 2015;7(1):26-26. doi:10.1186/s13073-015-0149-z

12. Kiliszek M, Burzynska B, Michalak M, et al. Altered gene expression pattern in peripheral blood mononuclear cells in patients with acute myocardial infarction. *PLoS One*. 2012;7(11):e50054-e50054. doi:10.1371/journal.pone.0050054

13. Davis S, Meltzer PS. GEOquery: a bridge between the Gene Expression Omnibus (GEO) and BioConductor. eng. *Bioinformatics (Oxford, England)*. 2007;23(14):1846-7.

14. Ritchie ME, Phipson B, Wu D, et al. limma powers differential expression analyses for RNA-sequencing and microarray studies. *Nucleic Acids Res*. 2015;43(7):e47. doi:10.1093/nar/gkv007

15. Sherman BT, Hao M, Qiu J, et al. DAVID: a web server for functional enrichment analysis and functional annotation of gene lists (2021 update). *Nucleic Acids Res*. 2022:gkac194. doi:10.1093/nar/gkac194

16. Liang B, Liang Y, Li R, Zhang H, Gu N. Integrating systematic pharmacology-based strategy and experimental validation to explore the synergistic pharmacological mechanisms of Guanxin V in treating ventricular remodeling. *Bioorg Chem*. 2021/10/01/ 2021;115:105187. doi:10.1016/j.bioorg.2021.105187

17. Zhang Q, Liu W, Zhang H-M, et al. hTFtarget: A Comprehensive Database for Regulations of Human Transcription Factors and Their Targets. *Genomics Proteomics Bioinformatics*. 2020;18(2):120-128. doi:10.1016/j.gpb.2019.09.006

18. Hu H, Miao Y-R, Jia L-H, Yu Q-Y, Zhang Q, Guo A-Y. AnimalTFDB 3.0: a comprehensive resource for annotation and prediction of animal transcription factors. *Nucleic Acids Res*. 2019;47(D1):D33-D38. doi:10.1093/nar/gky822

19. Szklarczyk D, Gable AL, Nastou KC, et al. The STRING database in 2021: customizable protein-protein networks, and functional characterization of user-uploaded gene/measurement sets. *Nucleic Acids Res*. 2021;49(D1):D605-D612. doi:10.1093/nar/gkaa1074

20. Liang B, Li R, Liang Y, Gu N. Guanxin V Acts as an Antioxidant in Ventricular Remodeling. Original Research. *Front Cardiovasc Med*. 2022-January-04 2022;8:778005. doi:10.3389/fcvm.2021.778005

21. Tang Y, Li M, Wang J-X, Pan Y, Wu F-X. CytoNCA: A cytoscape plugin for centrality analysis and evaluation of protein interaction networks. *Biosystems*. 2015/01/01/ 2015;127:67-72. doi:10.1016/j.biosystems.2014.11.005

22. Shannon P, Markiel A, Ozier O, et al. Cytoscape: a software environment for integrated models of biomolecular interaction networks. *Genome Res*. 2003;13(11):2498-2504.
